# Potential of terrestrial laser scanning for characterizing forest regeneration: A review and an experimental case study

**DOI:** 10.12688/openreseurope.21434.1

**Published:** 2025-11-06

**Authors:** Bojana Petrovic, Anna Candotti, Carlotta Grande, Miriam Sophie Faerber, Enrico Tomelleri

**Affiliations:** 1 Faculty of Forestry and Wood Technology, Mendel University, Brno, Czech Republic; 2 Faculty of Agricultural, Environmental and Food Sciences, Free University of Bozen-Bolzano, Bolzano, Italy; 3 Department of Environmental Engineering, Sapienza Universty of Rome, Rome, Italy

**Keywords:** Terrestrial laser scanning, Forest regeneration, post-disturbance recovery, Structural Forest metrics, Sentinel-2

## Abstract

Forest regeneration monitoring is essential for evaluating ecosystem recovery and informing sustainable forest management. Optical remote sensing methods such as NDVI and EVI are commonly used for this purpose, but they often fail to capture fine-scale structural information, particularly in early successional stages. Terrestrial Laser Scanning (TLS), in contrast, offers high-resolution, three-dimensional data capable of directly detecting tree structure, height distribution, and leaf area index (LAI). This study combines a systematic literature review with original TLS data collected from a regenerating forest plot affected by windstorm disturbance, where multispectral imagery was also acquired. The review highlights the advantages of TLS over optical techniques, particularly in detecting understory vegetation and capturing structural complexity. The experimental results support these findings: TLS enabled the accurate detection of tree saplings that were not visible in optical imagery and allowed for the extraction of height distributions and LAI. These results demonstrate that even single TLS acquisition can provide critical structural insights into forest regeneration that optical methods alone cannot deliver. We conclude that TLS is a valuable tool for monitoring post-disturbance recovery and advocates for its integration with optical remote sensing approaches such as NDVI and EVI to achieve a more comprehensive assessment of forest regeneration.

## Plain Language Summary

Forests naturally regrow after damage from storms or other events, but tracking this recovery is challenging, especially when trees are still small or hidden beneath larger plants. This study looked at how a technology called *terrestrial laser scanning* (TLS) can help. TLS uses ground-based lasers to create very detailed 3D maps of forest structure, capturing features like tree height, density, and leaf coverage. We reviewed recent research and also tested TLS in a forest in northern Italy damaged by a severe windstorm in 2018. The laser data were compared with satellite images that measure vegetation health through “greenness” indices like NDVI and EVI. TLS revealed small trees and undergrowth that the satellites could not detect, while the satellite images provided broader seasonal and yearly trends in plant growth. Our results show that using TLS alongside satellite data gives a fuller picture of forest recovery. This combined approach can help forest managers and conservationists make better decisions about post-storm recovery and long-term forest health.

## Introduction

Forest regeneration is a critical component of ecosystem recovery, particularly following disturbances such as windstorms, which can have long-term impacts and potentially severe consequences for ecosystem services ( Romagnoli *et al.*, 2023). Monitoring and quantifying forest regeneration plays a vital role in sustainable forest management and adaptive planning ( Miina *et al.*, 2006; Witzmann *et al.*, 2025). Traditionally, optical remote sensing techniques such as satellite and aerial imagery ( Lin *et al.*, 2024), multispectral sensors ( Panuju *et al.*, 2020), and vegetation indices like NDVI ( Huang *et al.*, 2021) have been widely applied to assess regeneration at broad spatial scales. However, these methods are limited in their ability to detect subcanopy structure, individual saplings, or early-stage regeneration ( Johnston *et al.*, 2018; Pontius *et al.*, 2020). Wind disturbance is currently the most significant natural disturbance affecting European forests ( Seidl *et al.*, 2014), causing widespread tree damage and influencing microclimatic conditions ( Palm-Hellenurm *et al.*, 2024). In mountain forests, such disturbances can result in long-lasting ecological consequences, including altered regeneration trajectories and shifts in forest composition ( Romagnoli *et al.*, 2023). One of the most impactful examples is Storm Vaia (October 27–30, 2018), which severely affected Northern Italy, damaging more than 42,500 hectares of forest with wind gusts exceeding 200 km/h ( Barbagallo *et al.*, 2024; Chirici *et al.*, 2019). Understanding natural regeneration in such contexts is essential for designing post-disturbance management strategies that foster ecosystem recovery and resilience ( Taeroe *et al.*, 2019). In recent years, TLS has become an important tool for detailed structural analysis of forests, producing high-resolution 3D point clouds that allow direct measurements of canopy height, stem density, leaf area index (LAI), and vertical profiles ( Liang *et al.*, 2016). TLS enables the detection of saplings, small trees, and even herbaceous layers ( Åkerblom & Kaitaniemi, 2021; Vicari *et al.*, 2019; Wilkes *et al.*, 2017). The aim of this study is to evaluate the potential of TLS in characterizing forest regeneration and to identify which structural characteristics can be detected with TLS but not with optical remote sensing methods. This paper combines a systematic literature review with original TLS and remote sensing data collected from a regenerating forest plot affected by Storm Vaia 2018, demonstrating the practical application of TLS for assessing structural features in forest recovery.

This study was guided by the following objectives:

1. To conduct a systematic literature review to determine the key structural characteristics of forest regeneration that are more accurately captured by terrestrial laser scanning (TLS) than by optical remote sensing methods.
2. To demonstrate the application of TLS through experimental field data to assess parameters such as tree segmentation, height distribution analysis, and leaf area index (LAI) in a regenerating forest plot.

## Material and methods

### Systematic Literature Review

A systematic literature review (SLR) was conducted through targeted searches in academic databases, including Scopus, Web of Science, Research Gate, using keywords such as "LiDAR", "Terrestrial Laser Scanning", "Forest Regeneration", and "Optical Sensing". Each query consisted of different keywords, and related synonyms were combined through logical operators (i.e., “AND”, “OR”). To capture recent advancements in the field, the search was restricted to peer-reviewed articles published between 2020 and 2025. The key structural characteristics of forest regeneration between the application of TLS and optical remote sensing were identified based on the final sample of 34 articles from the total database of 68 papers after thorough reading.

### Experimental data collection and TLS sampling

TLS data were collected using a RIEGL ( RIEGL Laser Measurement Systems, 2023) ground-based scanner from multiple positions and processed in RiSCAN PRO (RIEGL, Austria) for cleaning, alignment, and georeferencing. The registered 3D point cloud was exported to CloudCompare version 2.12.4 ( GPL software, 2022) (64-bit), where a Cloth Simulation Filter (CSF) was applied to separate ground and vegetation points, producing a Digital Terrain Model (DTM) and an off-ground canopy point cloud. A scalar field derived from cloud-to-mesh (C2M) distance was used to quantify vegetation height, and its distribution was analysed with CloudCompare’s histogram tool. Vegetation <2 m was removed to exclude shrubs and ground clutter, focusing on tree canopy structure. Remaining canopy points were segmented into individual crowns using connected component labeling. Each cluster was visually inspected and colorized by height, with one representative tree selected for detailed vertical profile analysis and height distribution assessment. Field observations documented vegetation composition, sapling height, and regeneration density, supported by photographs to aid interpretation of TLS data. To complement the structural metrics, NDVI and EVI were derived from Sentinel-2 Level-2A imagery (2018–2025) in Google Earth Engine ( Gorelick *et al.*, 2017). These indices provided information on canopy greenness and photosynthetic activity, enabling an integrated assessment of forest structure and condition. This combined approach supports repeatable, multi-season monitoring of regeneration dynamics and post-disturbance recovery.

## Results

### Results from literature review

Across the literature, TLS proved more effective than optical remote sensing for capturing detailed structural attributes of regenerating forests, especially in early successional stages. Common TLS-derived metrics included canopy height (29 studies), individual tree detection and stem density (25), LAI (18), and vertical structure including subcanopy layers (21). Optical methods more often provided NDVI and related indices (31), broad canopy cover estimates (19), and vegetation health assessments (14). TLS offers direct structural measurements, while optical methods primarily yield indirect estimates from spectral data. Studies consistently report that TLS offers superior performance in detecting early-stage regeneration and subcanopy structure compared to optical methods. Table 1 compares regeneration parameters, showing TLS’s clear advantage in detecting fine-scale details such as saplings and subcanopy structure. The table highlights TLS's superior performance in capturing fine-scale structural details that are typically missed by optical sensors.

**Table 1.** Comparison of forest regeneration characteristics as measured by TLS and optical remote sensing techniques.

Regeneration Parameter	TLS	Optical RS	References
Canopy Structure	Direct measurement	Indirect estimation	Santos *et al.*, 2022; Wardius & Hein (2024),
Tree detection- Stem density	High accuracy, including saplings	Limited, often missed in dense canopy	Bogdanovic *et al.*, 2021; Calders *et al.*, 2020; Wardius & Hein, 2024
LAI	Calculated from structural data	Indirect estimation via NDVI or models	Tian *et al.*, 2025; Wang & Fang, 2020; Wei *et al.*, 2020
NDVI -Vegetation Indices	Not applicable	Commonly used for spectral data	Liang *et al.*, 2025; Montero *et al.*, 2023
Vertical structure	Full 3D profile, including multiple layers	Limited to upper canopy	Muumbe *et al.*, 2021; Schraik *et al.*, 2023
Understory-Subcanopy detection	Clearly captured with high detail	Rarely detected below canopy	Adhikari *et al.*, 2023; Puletti *et al.*, 2021

### Results from experimental plot

***Tree segmentation, height distribution analysis and CHM***

**Figure 1.**
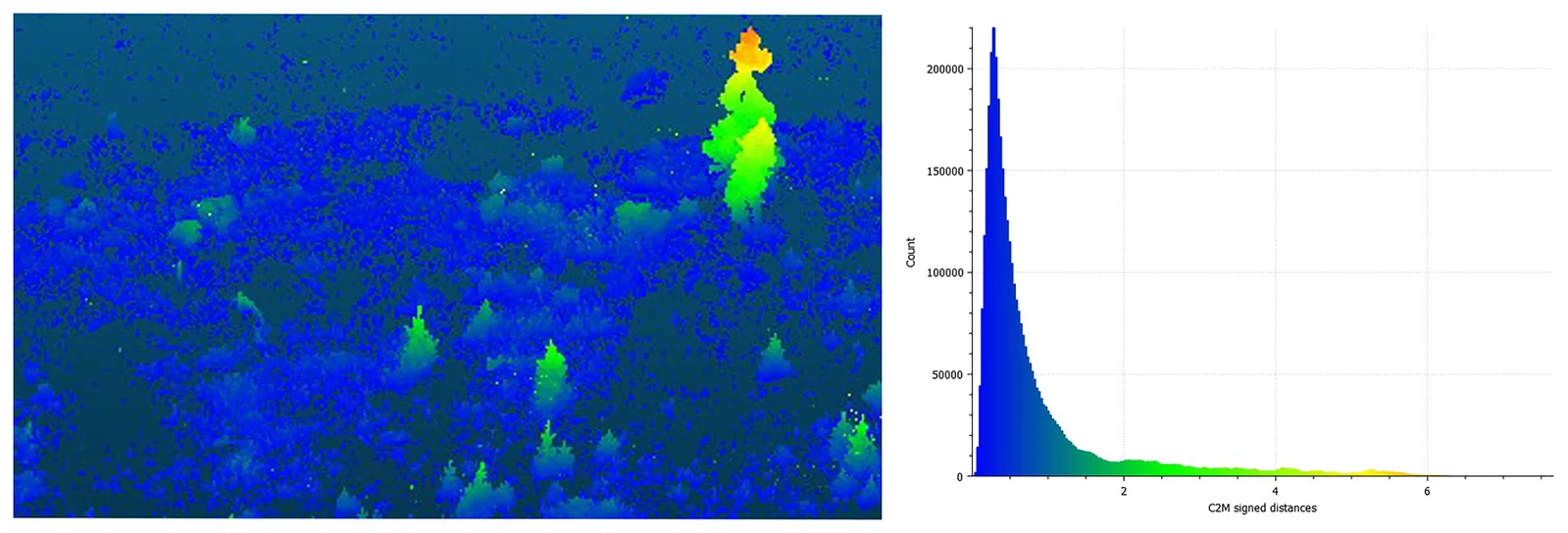
Left: Point cloud colorized by C2M signed distance showing above-ground vegetation structure. Right: Canopy height distribution histogram, showing the frequency of point heights derived from the C2M model.

**Figure 2.**
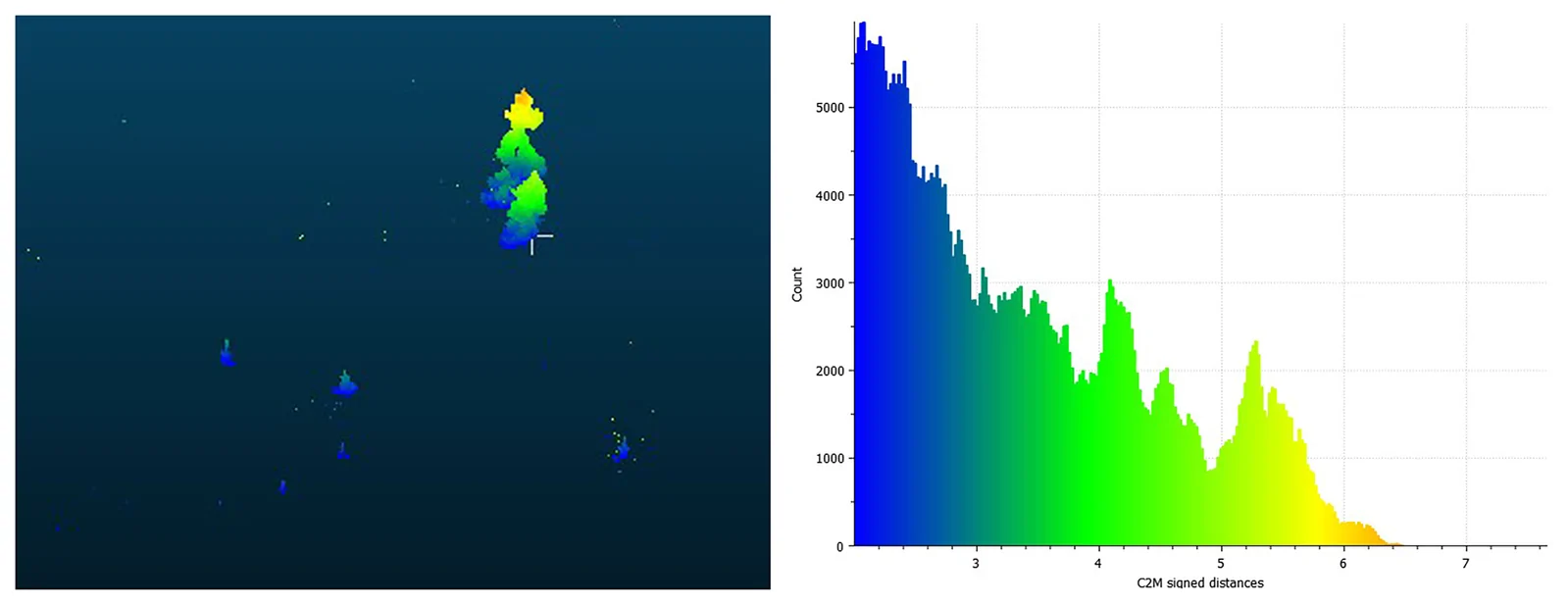
Individual tree extracted from TLS point cloud using connected component segmentation. Left: tree crown point cloud colorized by height (C2M signed distance). Right: vertical distribution of points within the tree, representing the canopy structure and height range.

The TLS-derived point cloud revealed detailed information on forest structure at both stand and individual tree levels. As shown in Figure 1, the colorized point cloud illustrates substantial spatial variability in canopy height across the plot, with isolated taller individuals emerging from a predominantly low-stature vegetative matrix. The accompanying histogram confirms a highly skewed height distribution, with the majority of points concentrated below 2 meters typical of early regeneration stages. In contrast, Figure 2 focuses on a segmented individual tree extracted using connected component analysis. The 3D point cloud reveals a well-defined vertical profile, while its height distribution shows multiple peaks, suggesting structural differentiation within the canopy.

**Figure 3.**
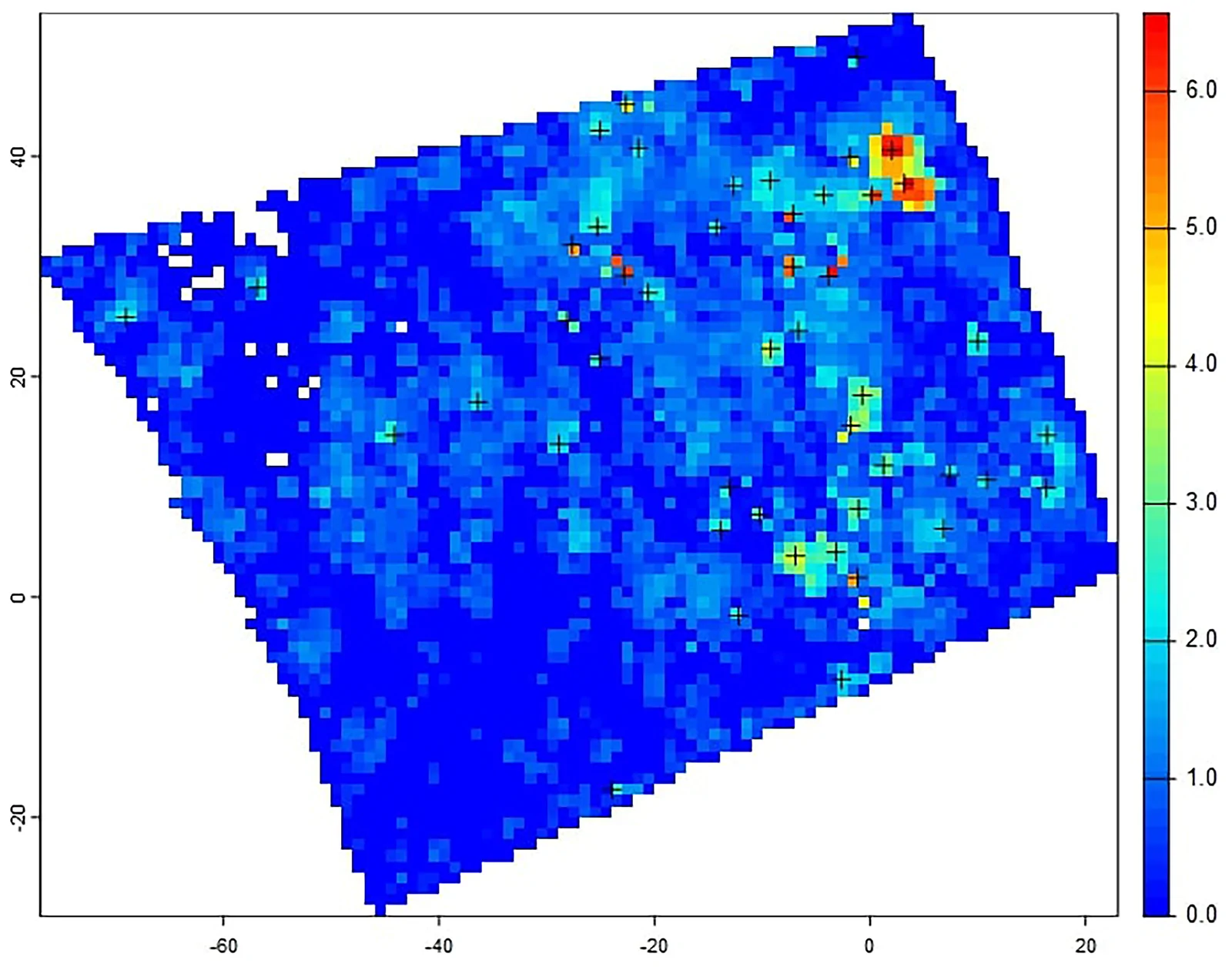
CHM generated from TLS data, showing canopy height (in meters) and detected individual treetops (black crosses) based on local maxima analysis.

Figure 3 shows CHM generated from TLS data, presented as a rasterized grid. The color gradient represents vegetation height in meters, with blue indicating lower vegetation and red representing taller canopy structures. Black cross markers (+) denote the locations of treetops identified using a local maximum filter applied to the CHM. This spatial representation reveals pronounced heterogeneity in vertical structure across the regenerating plot, with clusters of taller individuals embedded within a predominantly low-stature matrix. The CHM not only supports visualization of canopy development but also enables tree counting and spatial pattern analysis, contributing to a more detailed assessment of regeneration dynamics.

***Spatial and temporal patterns in vegetation indices***

**Figure 4.**
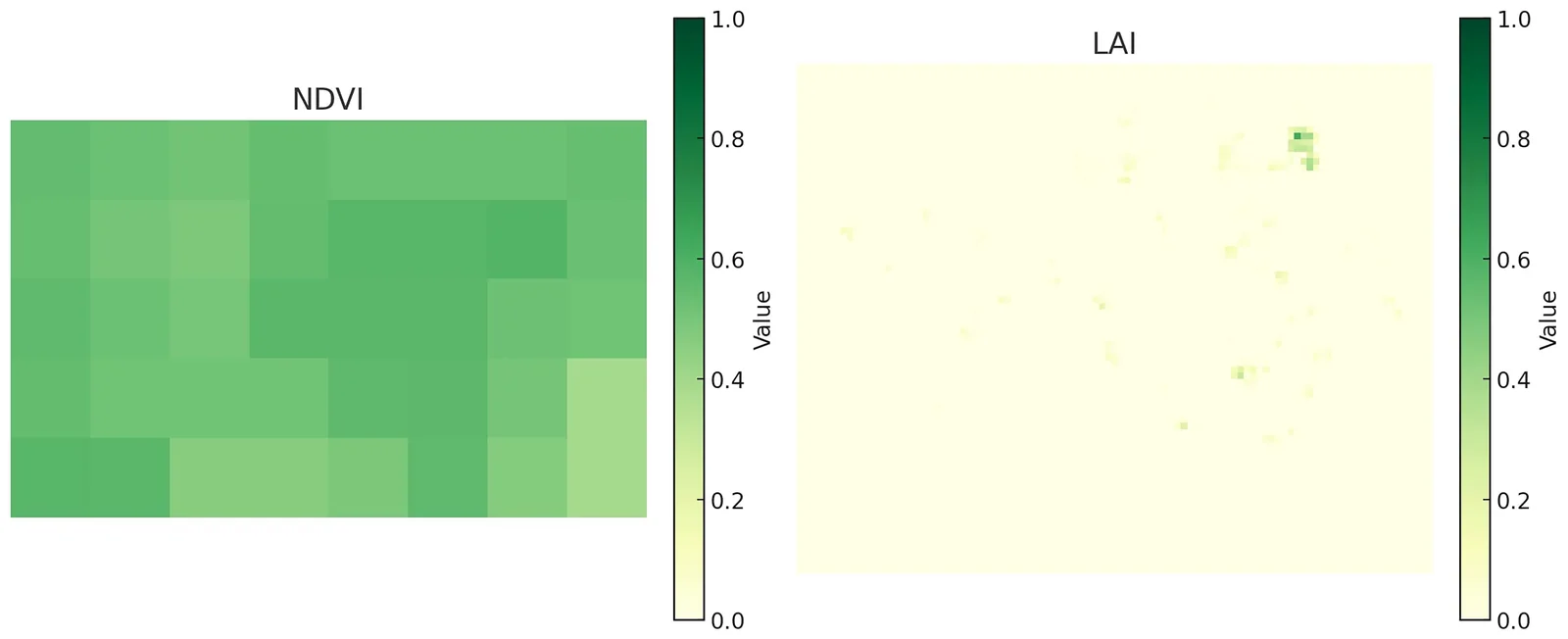
Mapped distribution of NDVI and LAI across the study area in 2025. The left panel shows NDVI (0–1) and the right panel shows LAI (0–6), derived from remote sensing data.

**Figure 5.**
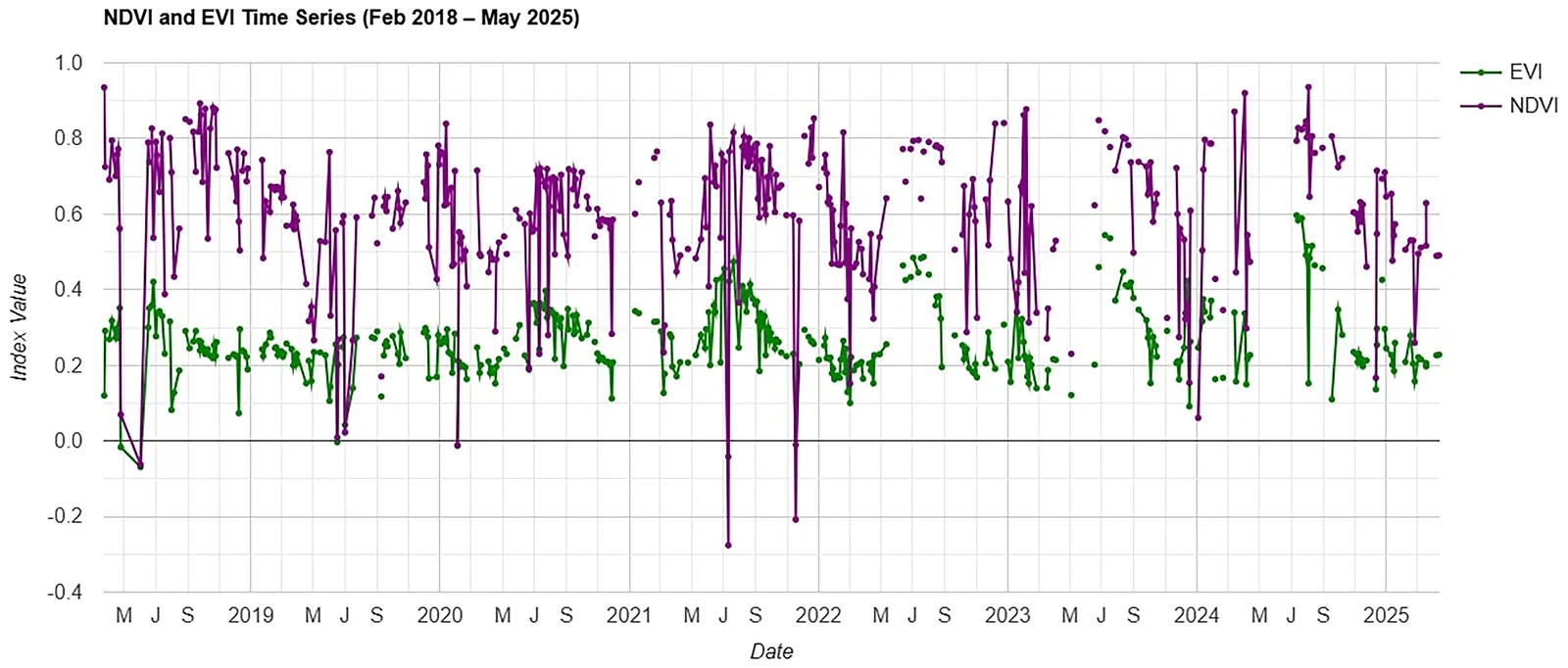
Time series of NDVI and EVI for the study area.

In 2025, NDVI values (0–1) reflected vegetation greenness, while LAI values (0–6) indicated canopy density ( Figure 4). Both maps showed high spatial heterogeneity, with patches of dense, green canopy interspersed among sparsely vegetated areas typical of early-stage regeneration. Time-series analysis of NDVI and EVI (2018–2025) revealed clear seasonal cycles, with peaks in the growing season and lows in winter ( Figure 5). NDVI was consistently higher, reflecting greater sensitivity to canopy greenness, whereas EVI was more conservative due to reduced atmospheric and soil background effects.

Interannual variation was linked to climate, phenology, and disturbance events, with occasional outliers from residual cloud or atmospheric noise. Together, these indices provided complementary insights into spatial structure and temporal dynamics when integrated with TLS measurements.

## Conclusion

This study highlights the value of TLS as a high-resolution tool for monitoring forest regeneration, particularly in structurally complex or early successional stands. Our literature review and case study show that TLS captures canopy height, stem density, and vertical structure often missed by optical remote sensing. The integration of TLS-derived structural metrics with spectral indices such as NDVI and EVI allowed for a more comprehensive assessment of vegetation condition, capturing both physiological and architectural aspects of forest recovery. Our findings support the growing body of evidence advocating for the use of TLS in forest ecology and suggest that combining structural and spectral data can enhance the accuracy and interpretability of regeneration assessments. TLS should be considered a complementary component in multi-source monitoring strategies aimed at evaluating post-disturbance forest dynamics and informing sustainable forest management.

## Ethics and consent

Ethical approval and consent were not required.

## Data Availability

The datasets used in this study, including NDVI and LAI raster files and TLS point-cloud data, are openly available on Zenodo: https://doi.org/10.5281/zenodo.15762119 ( Petrovic *et al.*, 2025). The version used in this article is https://doi.org/10.5281/zenodo.17349048 ( Petrovic *et al.*, 2025; Version 3). All files are released under a Creative Commons Attribution 4.0 International (CC BY 4.0) licence and are freely accessible without login or embargo. The Zenodo repository contains all processed outputs of the study, including NDVI and LAI raster files (GeoTIFF format), NDVI and EVI time series data (CSV format), TLS point cloud data (LAS format), representative figures (JPEG), and a README file describing the workflow. For transparency and reproducibility, the Google Earth Engine script used for NDVI/EVI processing is archived in the repository as a standalone JavaScript file. All data and code can be downloaded directly from Zenodo.

## Software availability

Al software and scripts used in this study are openly available to ensure transparency and reproducibility.

### Remote sensing data processing

Google Earth Engine scripts for NDVI and EVI computation ( JavaScript API) are provided in the Zenodo repository ( https://doi.org/10.5281/zenodo.17349048) ( Petrovic *et al.*, 2025). Time series were generated using mean values across the area of interest at 10 m spatial resolution.

Data is available under the terms of the Creative Commons Attribution 4.0 International

### TLS data processing

RiSCAN PRO (RIEGL, Austria) was used for point cloud registration and georeferencing. CloudCompare v2.12.4 ( https://www.cloudcompare.org) was used for ground filtering, height calculations, tree segmentation, and visualization.

Data is available under the terms of the GNU General Public License (GPL)
