# Potential of terrestrial laser scanning for characterizing forest regeneration: A review and an experimental case study

**DOI:** 10.12688/openreseurope.21434.2

**Published:** 2026-04-16

**Authors:** Bojana Petrovic, Anna Candotti, Carlotta Grande, Miriam Sophie Faerber, Enrico Tomelleri

**Affiliations:** 1 Faculty of Forestry and Wood Technology, Mendel University, Brno, Czech Republic; 2 Faculty of Agricultural, Environmental and Food Sciences, Free University of Bozen-Bolzano, Bolzano, Italy; 3 Department of Environmental Engineering, Sapienza Universty of Rome, Rome, Italy

**Keywords:** Terrestrial laser scanning, Forest regeneration, post-disturbance recovery, Structural Forest metrics, Sentinel-2

## Abstract

Forest regeneration monitoring is essential for evaluating ecosystem recovery and informing sustainable forest management. Optical remote sensing methods such as NDVI and EVI are commonly used for this purpose, but they often fail to capture fine-scale structural information, particularly in early successional stages. Terrestrial Laser Scanning (TLS), in contrast, offers high-resolution, three-dimensional data capable of directly detecting tree structure, height distribution, and leaf area index (LAI). This study combines a systematic literature review with original TLS data collected from a regenerating forest plot affected by windstorm disturbance, where multispectral imagery was also acquired. The review highlights the advantages of TLS over optical techniques, particularly in detecting understory vegetation and capturing structural complexity. The experimental results support these findings: TLS enabled the accurate detection of tree saplings that were not visible in optical imagery and allowed for the extraction of height distributions and LAI. These results demonstrate that even single TLS acquisition can provide critical structural insights into forest regeneration that optical methods alone cannot deliver. We conclude that TLS is a valuable tool for monitoring post-disturbance recovery and advocates for its integration with optical remote sensing approaches such as NDVI and EVI to achieve a more comprehensive assessment of forest regeneration.

## Plain Language Summary

Forests naturally regrow after damage from storms or other events, but tracking this recovery is challenging, especially when trees are still small or hidden beneath larger plants. This study looked at how a technology called *terrestrial laser scanning* (TLS) can help. TLS uses ground-based lasers to create very detailed 3D maps of forest structure, capturing features like tree height, density, and leaf coverage. We reviewed recent research and also tested TLS in a forest in northern Italy damaged by a severe windstorm in 2018. The laser data were compared with satellite images that measure vegetation health through “greenness” indices like NDVI and EVI. TLS revealed small trees and undergrowth that the satellites could not detect, while the satellite images provided broader seasonal and yearly trends in plant growth. Our results show that using TLS alongside satellite data gives a fuller picture of forest recovery. This combined approach can help forest managers and conservationists make better decisions about post-storm recovery and long-term forest health.

## Introduction

Forest regeneration is a critical component of ecosystem recovery, particularly following disturbances such as windstorms, which can have long-term impacts and potentially severe consequences for ecosystem services ( Romagnoli *et al.*, 2023). Monitoring and quantifying forest regeneration plays a vital role in sustainable forest management and adaptive planning ( Miina *et al.*, 2006; Witzmann *et al.*, 2025). Traditionally, optical remote sensing techniques such as satellite and aerial imagery ( Lin *et al.*, 2024), multispectral sensors ( Panuju *et al.*, 2020), and vegetation indices like NDVI ( Huang *et al.*, 2021) have been widely applied to assess regeneration at broad spatial scales. However, these methods are limited in their ability to detect subcanopy structure, individual saplings, or early-stage regeneration ( Johnston *et al.*, 2018; Pontius *et al.*, 2020). Wind disturbance is currently the most significant natural disturbance affecting European forests ( Seidl *et al.*, 2014), causing widespread tree damage and influencing microclimatic conditions ( Palm-Hellenurm *et al.*, 2024). In mountain forests, such disturbances can result in long-lasting ecological consequences, including altered regeneration trajectories and shifts in forest composition ( Romagnoli *et al.*, 2023). One of the most impactful examples is Storm Vaia (October 27–30, 2018), which severely affected Northern Italy, damaging more than 42,500 hectares of forest with wind gusts exceeding 200 km/h ( Barbagallo *et al.*, 2024; Chirici *et al.*, 2019). Understanding natural regeneration in such contexts is essential for designing post-disturbance management strategies that foster ecosystem recovery and resilience ( Taeroe *et al.*, 2019). In recent years, TLS has become an important tool for detailed structural analysis of forests, producing high-resolution 3D point clouds that allow direct measurements of canopy height, stem density, leaf area index (LAI), and vertical profiles ( Liang *et al.*, 2016). TLS enables the detection of saplings, small trees, and even herbaceous layers ( Åkerblom & Kaitaniemi, 2021; Vicari *et al.*, 2019; Wilkes *et al.*, 2017). While previous reviews have comprehensively addressed TLS applications in mature forest inventory, biomass estimation, and canopy structure analysis ( Calders *et al*., 2020; Liang *et al*., 2016), the specific application of TLS for monitoring post-disturbance forest regeneration remains relatively underexplored. Regeneration monitoring presents distinct challenges that differentiate it from mature forest assessment: (i) the need to detect small-stature vegetation including saplings often below 2 m in height, (ii) high spatial heterogeneity characteristic of early successional stages, (iii) the presence of mixed vegetation types including woody regeneration, herbaceous cover, and coarse woody debris, and (iv) rapid temporal changes in structural complexity. These characteristics make regeneration monitoring particularly well-suited to TLS’s fine-scale structural detection capabilities, yet systematic comparison with traditional optical remote sensing approaches in this specific context has been limited. This study addresses this gap by combining a structured literature synthesis with empirical field data to evaluate the comparative advantages of TLS for characterizing post-disturbance regeneration dynamics.

The aim of this study is to evaluate the potential of TLS in characterizing forest regeneration and to identify which structural characteristics can be detected with TLS but not with optical remote sensing methods. This paper combines a systematic literature review with original TLS and remote sensing data collected from a regenerating forest plot affected by Storm Vaia 2018, demonstrating the practical application of TLS for assessing structural features in forest recovery.

This study was guided by the following objectives:

• To conduct a systematic literature review to determine the key structural characteristics of forest regeneration that are more accurately captured by terrestrial laser scanning (TLS) than by optical remote sensing methods.
• To demonstrate the application of TLS through experimental field data to assess parameters such as tree segmentation, height distribution analysis, and leaf area index (LAI) in a regenerating forest plot.

## Material and methods

### Systematic literature review

A systematic literature review (SLR) was conducted through targeted searches in academic databases, including Scopus, Web of Science, Research Gate, which were selected to cover a broad range of peer-reviewed literature in forestry and remote sensing.

The search strategy combined keywords related to laser scanning, forest regeneration, and optical sensing, including “terrestrial laser scanning”, “TLS”, “LiDAR”, “forest regeneration”, “understory”, “optical remote sensing”, “NDVI”, and “EVI”. Boolean operators (“AND”, “OR”) were used to combine terms, and only articles published between 2020 and 2025 were considered in order to capture recent methodological developments.

The initial search returned 68 records ( Table 1). After removal of duplicates and screening of titles and abstracts, 52 studies were retained for further evaluation. Studies were retained if they (i) focused on forest regeneration or early successional stages, (ii) applied TLS and/or optical remote sensing methods to characterize structural or canopy attributes, and (iii) were published in peer-reviewed journals. Studies were excluded if they addressed unrelated applications (e.g. urban environments, infrastructure) or did not report structural forest metrics relevant to regeneration.

**Table 1.** Overview of the literature selection and inclusion process.

Selection stage	Criteria applied	Records (n)
Initial search	Database search: Scopus, Web of Science, ResearchGate Years: 2020–2025	68
Title/Abstract screening	Document type: Peer-reviewed articles Topic relevance: Forest + TLS + regeneration	52
Full-text assessment	Inclusion: Field data, forest ecosystems, TLS structural metrics Exclusion: No field validation, non-forest, no quantitative comparison	34
Final synthesis	Complete assessment of TLS vs optical methods for regeneration	34

**Table 2.** Comparison of forest regeneration characteristics as measured by TLS and optical remote sensing techniques.

Regeneration parameter	TLS	Optical RS	References
Canopy Structure	Direct measurement	Indirect estimation	Santos *et al.*, 2022; Wardius & Hein (2024)
Tree detection- Stem density	High accuracy, including saplings	Limited, often missed in dense canopy	Bogdanovic *et al.*, 2021; Calders *et al.*, 2020; Wardius & Hein, 2024
LAI	Calculated from structural data	Indirect estimation via NDVI or models	Tian *et al.*, 2025; Wang & Fang, 2020; Wei *et al.*, 2020
NDVI -Vegetation Indices	Not applicable	Commonly used for spectral data	Liang *et al.*, 2025; Montero *et al.*, 2023
Vertical structure	Full 3D profile, including multiple layers	Limited to upper canopy	Muumbe *et al.*, 2021; Schraik *et al.*, 2023
Understory-Subcanopy detection	Clearly captured with high detail	Rarely detected below canopy	Adhikari *et al.*, 2023; Puletti *et al.*, 2021

**Figure 1.**
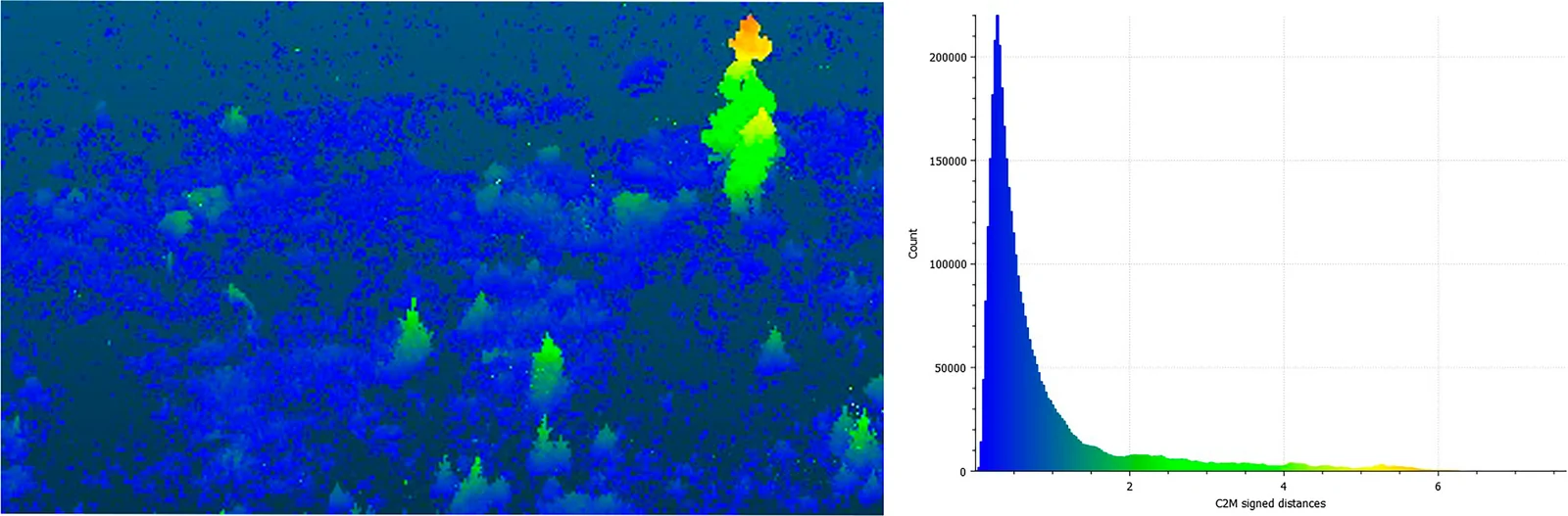
Left: Point cloud colorized by C2M signed distance showing above-ground vegetation structure. Right: Canopy height distribution histogram, showing the frequency of point heights derived from the C2M model.

Following full-text screening, 34 articles met the inclusion criteria and were retained for qualitative synthesis. From these studies, data were systematically extracted on study location, forest type, disturbance context, TLS specifications, derived metrics, and comparative performance with optical methods. Key regeneration-related structural parameters were extracted and compared, including canopy height, stem density, leaf area index (LAI), vertical structure, and understory detection, in order to assess the relative strengths of TLS and optical remote sensing approaches.

### Experimental data collection and TLS sampling

### Study area

A TLS survey was conducted in May 2025 in a regenerating forest near Nova Levante, Bolzano, Italy (46.42190° N, 11.57144° E), an area impacted by Storm Vaia in 2018. The site lies in a humid continental climate (Dfb, Köppen classification) with an average annual temperature of 5.5°C and annual precipitation of 1,022 mm. Dominant tree species include Norway spruce ( *Picea abies*), European larch ( *Larix decidua*), and Swiss stone pine ( *Pinus cembra*). The study plot was established to assess early forest recovery following windthrow disturbance, with TLS used to measure sapling density, height distribution, and structural complexity.

### TLS data acquisition

TLS data were acquired using a RIEGL VZ-600i terrestrial laser scanner (RIEGL Laser Measurement Systems GmbH, Horn, Austria), which operates at a 1550 nm wavelength and utilizes time-of-flight ranging with short infrared laser pulses. The scanner features a beam divergence of 0.3 mrad and offers a full 360° horizontal and 105° vertical field of view, enabling nearly spherical coverage from each scan position. Point cloud acquisition was performed using high-resolution scan settings (6 mm point spacing at 10 m distance) with a pulse repetition rate of up to 2.2 MHz, allowing for rapid data capture across varying terrain and vegetation structures. Multiple scan positions were established across the plot to ensure complete coverage and minimize occlusion effects from dense vegetation and terrain variability.

### Point cloud registration and preprocessing

Multiple scan positions were co-registered and georeferenced using RiSCAN PRO software (RIEGL Laser Measurement Systems, Austria). Point clouds from individual scans were cleaned to remove noise, outliers, and non-vegetation objects, then aligned and merged into a unified coordinate system. The registered 3D point cloud was exported to CloudCompare version 2.12.4 (64-bit, GPL software) for subsequent structural analysis.

### Ground classification and height normalization

To separate terrain from vegetation, a Cloth Simulation Filter (CSF) was applied within CloudCompare. This algorithm simulates a cloth draped over the inverted point cloud to identify ground points, which were then used to create a Digital Terrain Model (DTM). Off-ground points were retained for canopy analysis. Vegetation height was calculated as the cloud-to-mesh (C2M) signed distance between each vegetation point and the DTM surface. The resulting scalar field was analyzed using CloudCompare’s histogram tool, providing a height distribution of all vegetation in the plot. This distribution was used to assess vertical structure and to establish a 2 m threshold for filtering low vegetation, which typically includes shrubs, herbaceous cover, and ground clutter.

### Tree segmentation and individual crown

After filtering out points below 2 m height, the remaining canopy points were segmented into individual tree crowns using connected component labeling in CloudCompare. The “Label Connected Components” tool grouped spatially continuous points into discrete clusters, each representing a potential tree individual. Segmented clusters were visually inspected and colorized by height. One representative tree was selected for detailed vertical profile analysis, and its height distribution was visualized to assess crown structural complexity.

Field observations documented vegetation composition, sapling height, and regeneration density, supported by photographs to aid interpretation of TLS data. NDVI, EVI, and LAI were derived from Sentinel-2 Level-2A surface reflectance imagery using Google Earth Engine. LAI was estimated from NDVI using empirical relationships previously established for temperate coniferous forests ( Propastin & Panferov, 2013; Wang *et al*., 2005). These optical indices provided complementary information on vegetation greenness and canopy density alongside the three-dimensional structural information obtained from TLS.

## Results

### Results from literature review

Across the literature, TLS proved more effective than optical remote sensing for capturing detailed structural attributes of regenerating forests, especially in early successional stages. Common TLS-derived metrics included canopy height (29 studies), individual tree detection and stem density (25), LAI (18), and vertical structure including subcanopy layers (21). Optical methods more often provided NDVI and related indices (31), broad canopy cover estimates (19), and vegetation health assessments (14). TLS offers direct structural measurements, while optical methods primarily yield indirect estimates from spectral data. Studies consistently report that TLS offers superior performance in detecting early-stage regeneration and subcanopy structure compared to optical methods. Table 2 compares regeneration parameters, showing TLS’s clear advantage in detecting fine-scale details such as saplings and subcanopy structure. The table highlights TLS’s superior performance in capturing fine-scale structural details that are typically missed by optical sensors.

### Results from experimental plot

### Tree segmentation, height distribution analysis and CHM

**Figure 2.**
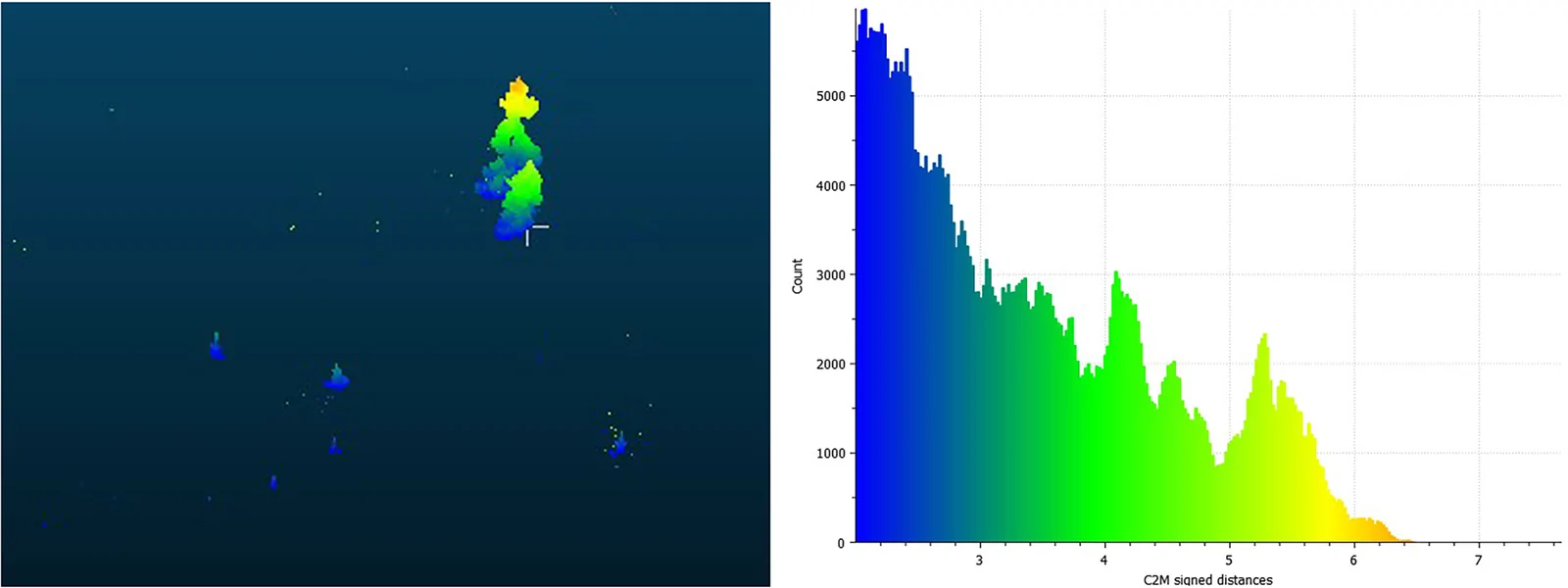
Individual tree extracted from TLS point cloud using connected component segmentation. Left: tree crown point cloud colorized by height (C2M signed distance). Right: vertical distribution of points within the tree, representing the canopy structure and height range.

The TLS-derived point cloud revealed detailed information on forest structure at both stand and individual tree levels. As shown in Figure 1, the colorized point cloud illustrates substantial spatial variability in canopy height across the plot, with isolated taller individuals emerging from a predominantly low-stature vegetative matrix. The accompanying histogram confirms a highly skewed height distribution, with the majority of points concentrated below 2 meters typical of early regeneration stages. In contrast, Figure 2 focuses on a segmented individual tree extracted using connected component analysis. The 3D point cloud reveals a well-defined vertical profile, while its height distribution shows multiple peaks, suggesting structural differentiation within the canopy.

**Figure 3.**
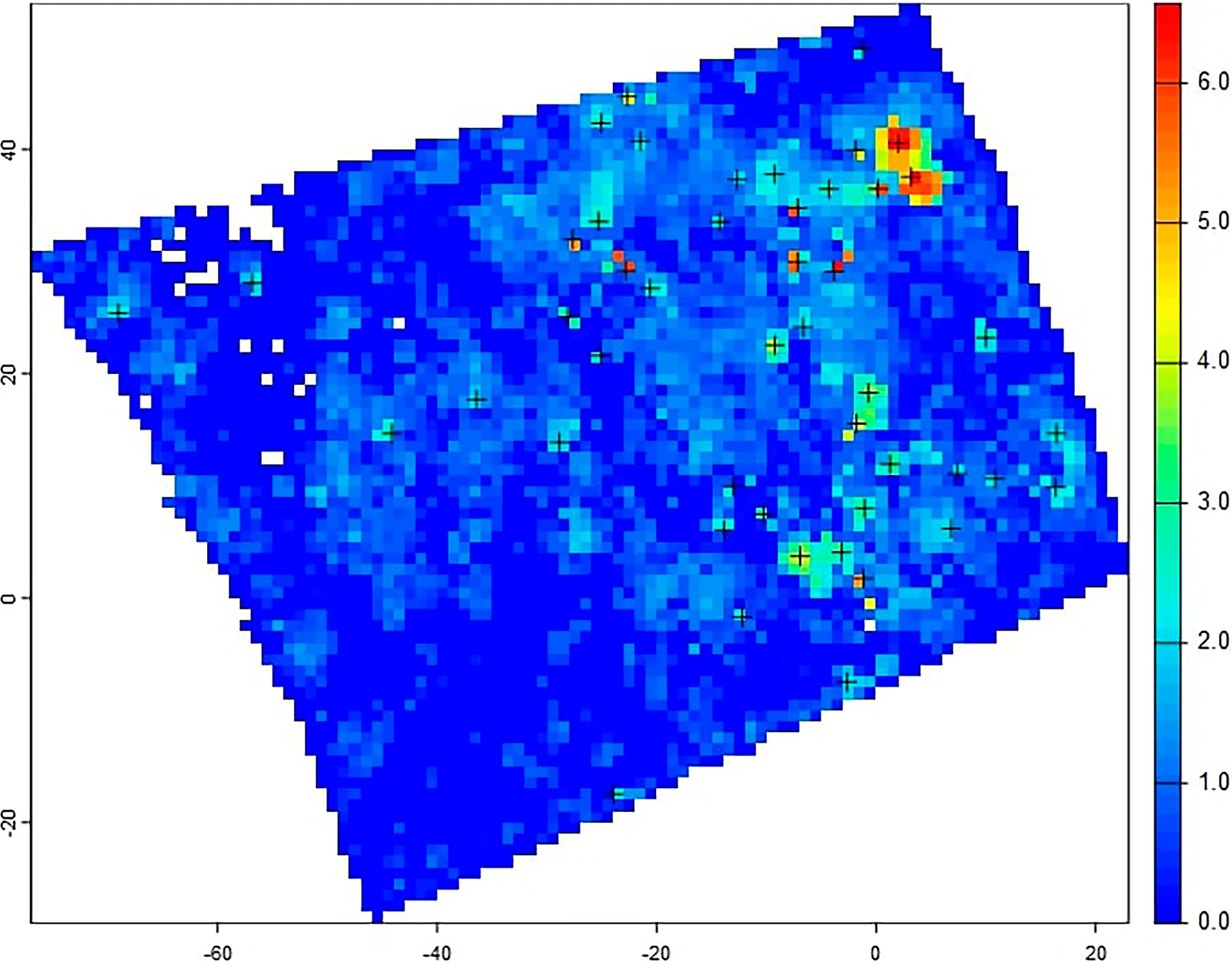
CHM generated from TLS data, showing canopy height (in meters) and detected individual treetops (black crosses) based on local maxima analysis.

Figure 3 shows CHM generated from TLS data, presented as a rasterized grid. The color gradient represents vegetation height in meters, with blue indicating lower vegetation and red representing taller canopy structures. Black cross markers (+) denote the locations of treetops identified using a local maximum filter applied to the CHM. This spatial representation reveals pronounced heterogeneity in vertical structure across the regenerating plot, with clusters of taller individuals embedded within a predominantly low-stature matrix. The CHM not only supports visualization of canopy development but also enables tree counting and spatial pattern analysis, contributing to a more detailed assessment of regeneration dynamics.

The quantitative contrasts in Table 3 reflect the structural characteristics observed in the study plot, where regeneration was dominated by saplings between 0.5 and 2.5 m in height. TLS enabled direct detection and segmentation of these individuals, whereas Sentinel-2 based optical indices provided spatially averaged information dominated by the upper vegetation signal. This illustrates why TLS is particularly suitable for characterizing early-stage forest regeneration, while optical remote sensing is better suited for broader-scale monitoring of canopy greenness and temporal trends.

**Table 3.** Quantitative comparison of terrestrial laser scanning (TLS) and optical remote sensing for forest regeneration assessment in the study plot.

Parameter	TLS (this study)	Optical remote sensing (Sentinel-2)
Spatial resolution	~6 mm point spacing at 10 m	10 m pixel size
Detectable vegetation height	Saplings ≥0.5 m	Dominant canopy signal only
Dominant regeneration height	0.5–2.5 m saplings clearly resolved	Height not directly resolved
Detection of individual saplings	Individual saplings segmented	Individual saplings not detectable
Sensitivity to vegetation <2 m	High (majority of points <2 m detected)	Very low
Height information	Direct 3D measurement	Indirect proxy
LAI estimation	Structure-based	Spectral index-based
Suitability for early regeneration	High	Limited

*Note:* Values are indicative for the single study plot and are intended to illustrate methodological differences rather than provide statistical validation.

**Figure 4.**
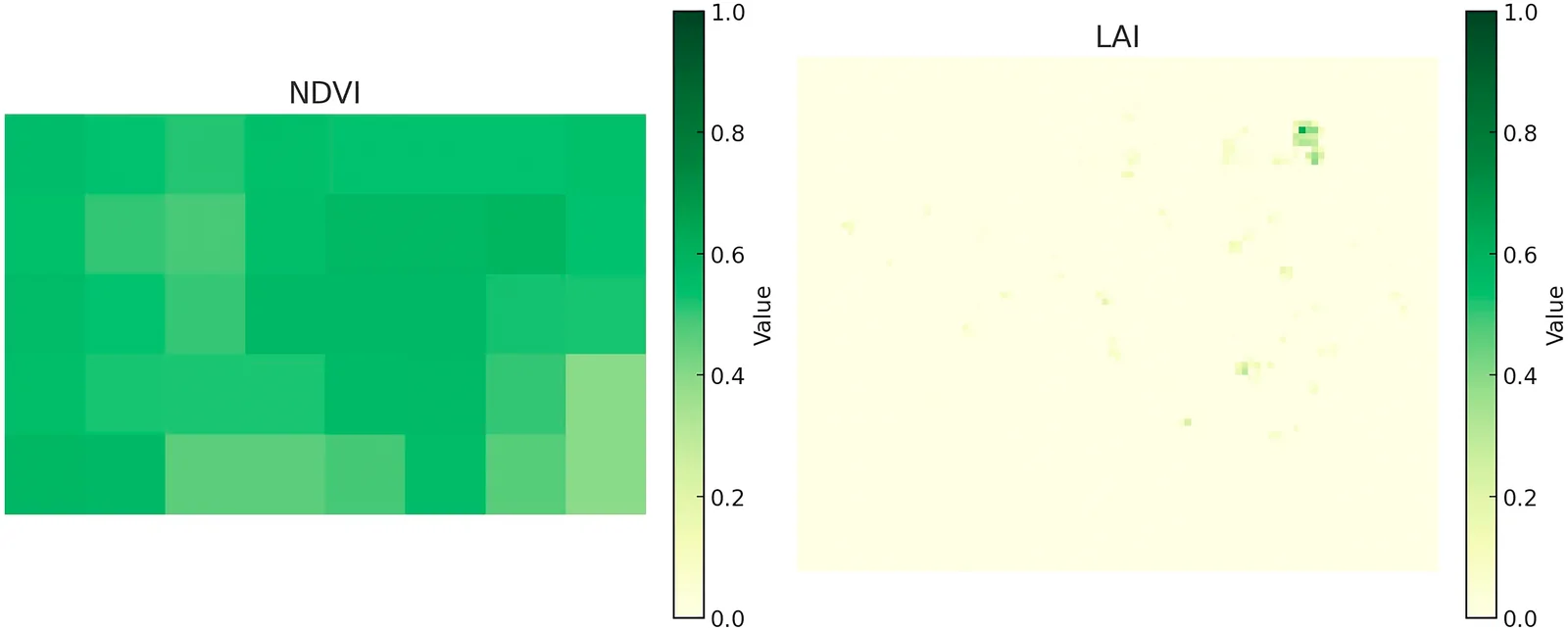
Mapped distribution of NDVI and LAI across the study area in 2025. The left panel shows NDVI (0–1) and the right panel shows LAI (0–6), derived from remote sensing data.

**Figure 5.**
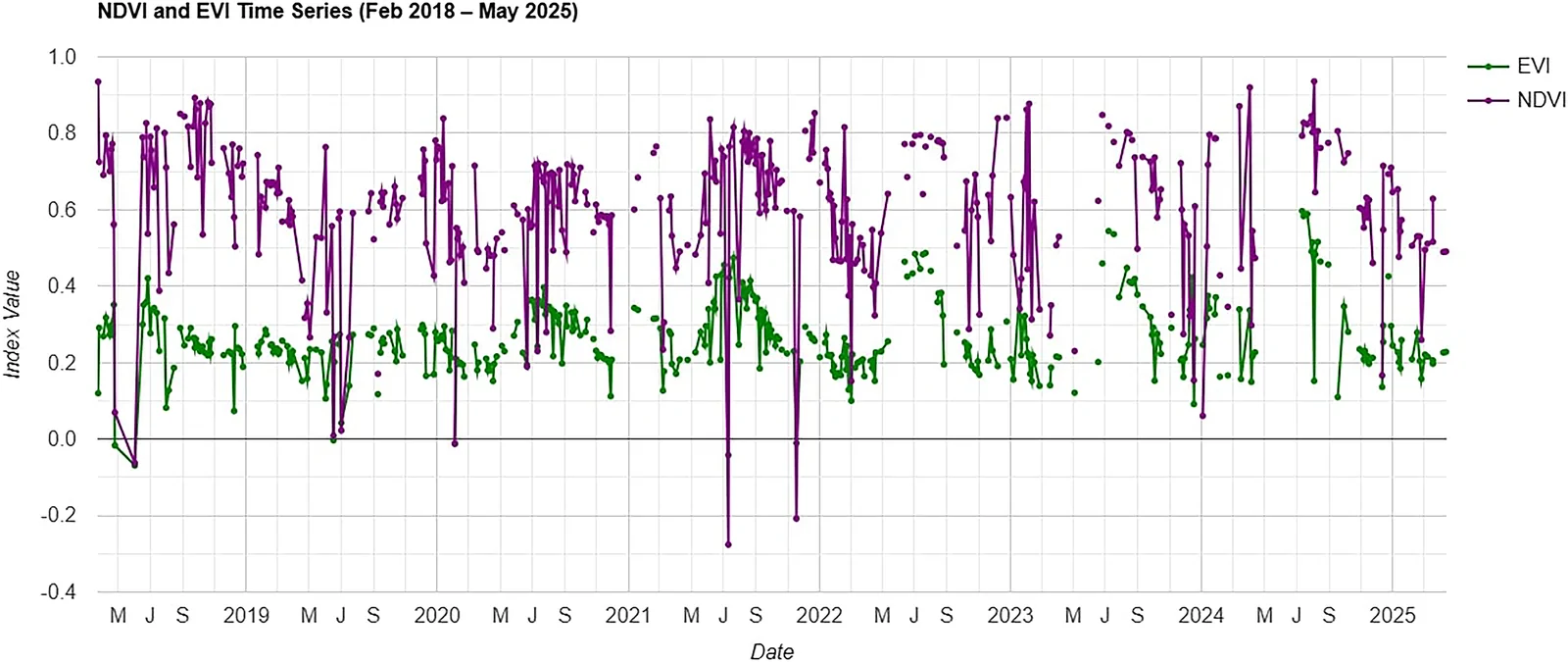
Time series of NDVI and EVI for the study area.

### Spatial and temporal patterns in vegetation indices

In 2025, NDVI values (0–1) reflected vegetation greenness, while LAI values (0–6) indicated canopy density ( Figure 4). Both maps showed high spatial heterogeneity, with patches of dense, green canopy interspersed among sparsely vegetated areas typical of early-stage regeneration. Time-series analysis of NDVI and EVI (2018–2025) revealed clear seasonal cycles, with peaks in the growing season and lows in winter ( Figure 5). NDVI was consistently higher, reflecting greater sensitivity to canopy greenness, whereas EVI was more conservative due to reduced atmospheric and soil background effects.

Interannual variation was linked to climate, phenology, and disturbance events, with occasional outliers from residual cloud or atmospheric noise. Together, these indices provided complementary insights into spatial structure and temporal dynamics when integrated with TLS measurements.

### Integration of TLS with optical remote sensing

To fully leverage the complementary strengths of TLS and optical remote sensing, we propose several concrete integration strategies for operational forest regeneration monitoring. First, TLS-derived structural metrics (tree height, crown dimensions, stem density, vertical profiles) can serve as high-fidelity reference data for upscaling and validating structural predictions from drone or satellite imagery at broader spatial scales ( Calders *et al.*, 2020). For example, plot-level TLS measurements of sapling height and density could be used to train random forest or neural network models that predict these parameters from multispectral drone imagery across entire watersheds. Second, TLS-derived 3D canopy structure can provide direct input to physically-based radiative transfer models (e.g., DART, librat) to improve retrieval of biophysical parameters such as LAI, fraction of absorbed photosynthetically active radiation (fAPAR), and chlorophyll content from satellite spectral data ( Gastellu-Etchegorry *et al*., 2015; Widlowski *et al*., 2013). Third, a multi-scale hierarchical approach combining plot-level TLS (~10 ^2^ m ^2^), landscape-level drone LiDAR (~10 ^4^–10 ^6^ m ^2^), and regional satellite imagery (~10 ^6^–10 ^9^ m ^2^) offers a practical pathway for upscaling fine-scale regeneration processes to management-relevant extents while maintaining structural fidelity ( Duncanson *et al*., 2022). Drone-based LiDAR serves as a critical intermediate scale, bridging the resolution gap between TLS and satellite platforms ( Wallace *et al*., 2016). However, this multi-scale integration presents challenges including differences in sampling geometry, spatial resolution mismatches, and the need for robust upscaling algorithms that account for within-pixel heterogeneity in early-successional forests. Future research should focus on developing standardized protocols for TLS-to-satellite data fusion and quantifying uncertainty propagation across scales ( Calders *et al*., 2020).

### Limitations and challenges

Several limitations should be acknowledged. First, our case study was conducted in a single forest type- a mixed conifer forest dominated by Norway spruce, European larch, and Swiss stone pine limiting generalizability to other vegetation communities such as broadleaf forests, mixed temperate forests, or tropical systems. Additionally, the study focused on a single disturbance event (Storm Vaia 2018), and findings may not directly translate to other disturbance regimes such as fire, insect outbreaks, clear-cutting, or drought-induced mortality. Second, the single-epoch TLS survey provides a structural snapshot but cannot capture temporal dynamics of regeneration; based on observed sapling growth rates in similar post-windthrow sites, we recommend repeated TLS surveys at 2–3 year intervals to effectively monitor structural changes while balancing logistical constraints. Third, TLS data collection and processing are resource-intensive, requiring specialized equipment, trained personnel, and approximately 4–6 hours of field time per plot, compared to freely available satellite imagery. Fourth, while TLS excels at capturing fine-scale structure, it lacks the spectral information needed for species identification or physiological assessment without complementary data sources. Fifth, TLS performance may decline in extremely dense regeneration where occlusion limits laser penetration, though this was not a major constraint in our early-successional plot. Finally, our study focused on plot-scale assessment (~400 m ^2^); scaling TLS-derived insights to landscape or regional levels would require integration with airborne LiDAR or satellite-based approaches.

## Conclusion

This study highlights the value of TLS as a high-resolution tool for monitoring forest regeneration, particularly in structurally complex or early successional stands. Our literature review and case study show that TLS captures canopy height, stem density, and vertical structure often missed by optical remote sensing. The integration of TLS-derived structural metrics with spectral indices such as NDVI and EVI allowed for a more comprehensive assessment of vegetation condition, capturing both physiological and architectural aspects of forest recovery. Our findings support the growing body of evidence advocating for the use of TLS in forest ecology and suggest that combining structural and spectral data can enhance the accuracy and interpretability of regeneration assessments. TLS should be considered a complementary component in multi-source monitoring strategies aimed at evaluating post-disturbance forest dynamics and informing sustainable forest management.

## Ethics and consent

Ethical approval and consent were not required.

## Data availability

The datasets used in this study, including NDVI and LAI raster files and TLS point-cloud data, are openly available on Zenodo: 10.5281/zenodo.15762119 ( Petrovic *et al.*, 2025). The version used in this article is 10.5281/zenodo.17349048 ( Petrovic *et al.*, 2025; Version 3). All files are released under a Creative Commons Attribution 4.0 International (CC BY 4.0) licence and are freely accessible without login or embargo. The Zenodo repository contains all processed outputs of the study, including NDVI and LAI raster files (GeoTIFF format), NDVI and EVI time series data (CSV format), TLS point cloud data (LAS format), representative figures (JPEG), and a README file describing the workflow. For transparency and reproducibility, the Google Earth Engine script used for NDVI/EVI processing is archived in the repository as a standalone JavaScript file. All data and code can be downloaded directly from Zenodo.

## Software availability

Al software and scripts used in this study are openly available to ensure transparency and reproducibility.

### Remote sensing data processing

Google Earth Engine scripts for NDVI and EVI computation ( JavaScript API) are provided in the Zenodo repository ( 10.5281/zenodo.17349048) ( Petrovic *et al.*, 2025). Time series were generated using mean values across the area of interest at 10 m spatial resolution.

Data is available under the terms of the Creative Commons Attribution 4.0 International.

## TLS data processing

RiSCAN PRO (RIEGL, Austria) was used for point cloud registration and georeferencing. CloudCompare v2.12.4 ( https://www.cloudcompare.org) was used for ground filtering, height calculations, tree segmentation, and visualization.

Data is available under the terms of the GNU General Public License (GPL).

## Authors’ contributions

All authors contributed to the project’s conceptualization, methodology, data collection, and visualization. B. P contributed to TLS data collection, analysis, and manuscript writing. A. C and C. G contributed to data analysis and interpretation. M.S.F supported visualization and manuscript review. E. T supervised the project. All authors approved the final manuscript.
