# Supplementary material for: Potential of terrestrial laser scanning for characterizing forest regeneration: A review and an experimental case study
Source: Open Res Eur. 2026 Apr 16;5:341. Originally published 2025 Nov 6. [Version 2] doi: 10.12688/openreseurope.21434.2 (PMC13560847; doi:10.12688/openreseurope.21434.2)
Supplement: Peer review history

# Reviewer report: Reviewer response for version 2

**DOI:** 10.21956/openreseurope.25348.r77969

**Published:** 2026-08-21

**Authors:** Marc S. Adams

**Affiliations:** 1 Austrian Research Centre for Forests (BFW), Innsbruck, Austria

***General comments***

 In this contribution, the merits of Terrestrial Laser Scanning (TLS) for capturing key structural characteristics of forest regeneration on a wind-disturbed plot in Italy are discussed. Additionally, the article includes a systematic literature review to determine which key characteristics are relevant. Thirdly, the manuscript includes a juxtaposition of the potential of TLS and Sentinel-2-based vegetation indies (i.e. NDVI and EVI).

 On the whole, I found the first two topics mentioned above relevant to the field and worthy of scientific inquiry. The manuscript provides a well-structured basis for this kind of inquiry. However, the third point, the direct comparison of TLS (point spacing 6 mm) with vegetation indices derived from Sentinel-2 satellite data with 10 m ground sampling distance, seemed to invite a foregone conclusion, as one would hardly expect such spatially coarse data as Sentinel-2 of being capable of detecting fine-scale details such as saplings and subcanopy structure. I’m therefore having some trouble getting on board with what I perceive to be one of the main selling points of this article, i.e. that such a comparison is warranted and requires detailed investigation. The results presented in the manuscript are somewhat vague as little direct connection I could see was made between TLS and Sentinel.2 results. Furthermore, it seemed to me that the discussion at the end of the manuscript sounded more like a somewhat general reflection of potentially interesting (further) related topics than a being directly linked to the results presented in this article.

 Overall, the article is well written and comprehensible. Some of the figures (e.g. figure 1 and 2) could be double-checked for font size to improve legibility. I found some minor typos, which should be checked for by a type-setter. The majority of the figures are well presented and designed.

***Specific comments***

 Reading the abstract and the plain language summary, I had the impression that some important information was missing for the former (i.e. the type of optical remote sensing data used, the location where the data was collected, etc.). I would advise rephrasing these two paragraphs in such a way that they at bottom include the same information. For consistency, I advise adding the explanation of the acronyms NDVI and EVI (analogue to TLS and LAI) to the abstract. In the abstract and throughout the manuscript a variety of synonyms for Sentinel-2 data and indices derived thereof are used (optical remote sensing, multispectral imagery, optical techniques, optical imagery, etc.), which was somewhat confusing to me, as it kept me wondering, which data and remote sensing platform were actually included in the investigation. This was only cleared up on page 5 of the manuscript. Mentioning Sentinel-2 as the main dataset and adding a brief description early on would in my opinion add clarity to the manuscript.

 Along these lines, with some of the comments and conclusions in the manuscript I was unsure whether these referred to optical remote sensing in general or Sentinel-2 in particular (e.g. in the subsections ‘Results from literature review’ or ‘Results from experimental plot’). I would advise rephasing these sections and more clearly point out, which remote sensing data or method is referred to.

 In the chapter ‘Introduction’ I tripped over the “most significant natural disturbance”. It would seem to me to be clearer to speak of abiotic rather than natural disturbances, but this might be a matter of taste.

 Overall, I missed having at least a short description and small figure of the study site for which data was collected. The result plots shown in the figures seemed to change in viewpoint and extent of the study site thus in my opinion reducing comprehensibility and comparability of the results.

 In the same vein: I could not make out how the left sub-figure of figure 4 was connected to the rest of the presented results. If I understood correctly, LAI was derived both from Sentinel-2 and TLS? If this was so, which of the two is presented on the right of Figure 4? Or could the two LAI results maybe benefit from a direct juxtaposition in this figure?

 The subchapter ‘Spatial and temporal patterns in vegetation indices’ starts with ‘In 2025’, which seems to me could be omitted.

 The manuscript underscores the importance and potential of integrating TLS and Sentinel-2 data and results, but I could not find any attempt at doing this with the results from this study. It would seem to me to be a good starting point, since all necessary data and results seem to be at hand.

 In the subchapter ‘Integration of TLS with optical remote sensing’ several other remote sensing techniques are mentioned, which would in theory be available for multi-scale integration. I have the impression this section would be better suited to an outlook chapter at the very end of the manuscript.

Is the study design appropriate and does the work have academic merit?

Partly

Is the work clearly and accurately presented and does it cite the current literature?

Yes

If applicable, is the statistical analysis and its interpretation appropriate?

Yes

Are all the source data underlying the results available to ensure full reproducibility?

Yes

Are the conclusions drawn adequately supported by the results?

Partly

Are sufficient details of methods and analysis provided to allow replication by others?

Yes

Reviewer Expertise:

close-range and remote sensing

I confirm that I have read this submission and believe that I have an appropriate level of expertise to confirm that it is of an acceptable scientific standard, however I have significant reservations, as outlined above.

# Reviewer report: Reviewer response for version 1

**DOI:** 10.21956/openreseurope.23183.r69638

**Published:** 2026-03-09

**Authors:** Ljiljana Brasanac-Bosanac

**Affiliations:** 1 Institute of Forestry, Belgrade, Serbia

The title of the article is suitable. I don’t have any complaint.

 The manuscript is very interesting, it deals with current issues, especially from the aspect of the sustainable forest management. It has a scientific contribution as it details the importance of remote sensing. 

 The manuscript highlights the value of TLS as a high-resolution tool for monitoring forest regeneration, particularly in structurally complex or early successional stands which is very important for future researches.

 The scientific content of this study is well demonstrated in the paper.

 The language/English quality of article is suitable.

Is the study design appropriate and does the work have academic merit?

Yes

Is the work clearly and accurately presented and does it cite the current literature?

Yes

If applicable, is the statistical analysis and its interpretation appropriate?

I cannot comment. A qualified statistician is required.

Are all the source data underlying the results available to ensure full reproducibility?

Yes

Are the conclusions drawn adequately supported by the results?

Yes

Are sufficient details of methods and analysis provided to allow replication by others?

Yes

Reviewer Expertise:

Forestry

I confirm that I have read this submission and believe that I have an appropriate level of expertise to confirm that it is of an acceptable scientific standard.

# Response

**Published:** 2026-03-11

**Authors:** Bojana Petrovic

**Affiliations:** Department of Geology and Soil Science, Mendel University in Brno, Brno, South Moravian Region, Czech Republic

We are pleased that the reviewer found the topic relevant to sustainable forest management, acknowledged the scientific contribution of the work, and considered the content and language quality to be suitable for publication. As no revisions were requested by Reviewer 2, we have not made any changes in response to their comments. We hope that the revisions carried out in response to Reviewer 1 further strengthen the manuscript. Sincerely, Bojana Petrovic (Corresponding Author) bojana.petrovic@mendelu.cz

# Reviewer report: Reviewer response for version 1

**DOI:** 10.21956/openreseurope.23183.r64321

**Published:** 2025-12-24

**Authors:** Ewa Głowienka

**Affiliations:** 1 AGH University of Krakow, Krakow, Poland

The manuscript examines the use of terrestrial laser scanning for monitoring forest regeneration. The authors combine a structured review of published work with a field case in a stand damaged by wind. They compare information from terrestrial laser scanning with information from multispectral imagery and indices such as NDVI and EVI. The topic is important and current. Many groups still rely mainly on optical data for this type of monitoring, while the potential of terrestrial laser scanning in young stands and understorey is not fully clear.

 I find the general message convincing. The paper shows that terrestrial laser scanning can reveal fine structure and saplings that cannot be seen in optical images. The study design has clear merit and the link between the literature review and the case study is a strong point. At the same time, several parts of the text would benefit from more detail and clarification.

 First, the review part is called a systematic review, but the description of the procedure is brief. Readers should know which databases were used, which search terms and years were covered, and which criteria were used to include or exclude papers. It would also help to report how many papers were found at the start and how many remained after screening. A short table or a simple scheme would make this part transparent.

 Second, the paper could position itself more clearly with respect to existing reviews of terrestrial laser scanning in forests. Earlier work has already discussed applications in biomass, stand inventory and canopy structure. Here the focus is on regeneration after disturbance, which is a valuable and more narrow view. I suggest that the authors state more directly what is specific for regeneration, for example presence of saplings under partial canopy and strong small-scale variation, and how this work adds to the previous reviews.

 Third, the description of the terrestrial laser scanning data and processing in the case study is too short for full replication. The authors should provide more information on the scanner model, the main survey parameters, the number and layout of scan positions, and the registration procedure. The main processing steps also need a clearer description, including ground classification, segmentation of saplings and the way height and leaf area index were derived. A simple workflow from field survey to structural variables would be very useful.

 The comparison between terrestrial laser scanning and optical data is interesting, but mostly qualitative. If possible, I recommend that the authors add some simple numbers, for example the proportion of saplings detected by each method, basic error ranges for height or leaf area index, and more precise information on the optical platform and its resolution. Even a small table would make the advantage of terrestrial laser scanning more concrete.

 In the discussion, a compact paragraph on limitations would improve the balance of the paper. This could include the fact that the case study concerns one forest type and one disturbance, the known limits of terrestrial laser scanning in dense regeneration, and practical constraints such as cost and time in the field. The authors could also comment on how often such surveys would need to be repeated to follow regeneration in a useful way.

 Finally, the call for integration of terrestrial laser scanning and optical remote sensing is important, but at present rather general. I encourage the authors to add a few short and concrete examples, such as use of terrestrial laser scanning as a reference for training models for drone or satellite data, or as a source of structural input for radiative transfer models. A brief note on the problem of scale, and on possible use of drone-based data as an intermediate step, would also be helpful.

 Minor issues include consistent definition and use of abbreviations, a smoother transition between the review and the case study, and a check of figure readability and language. These are all manageable.

 In summary, I support the Indexing of this article, but I recommend that the authors address the points above, mainly by adding detail to the methods, clarifying the review procedure, and slightly expanding the discussion of limitations and integration with other data sources.

Is the study design appropriate and does the work have academic merit?

Yes

Is the work clearly and accurately presented and does it cite the current literature?

Partly

If applicable, is the statistical analysis and its interpretation appropriate?

Not applicable

Are all the source data underlying the results available to ensure full reproducibility?

Partly

Are the conclusions drawn adequately supported by the results?

Yes

Are sufficient details of methods and analysis provided to allow replication by others?

Partly

Reviewer Expertise:

Remote sensing of vegetation and forests, integration of structural and optical data, environmental applications of geospatial methods. Monitoring of forest regeneration and impacts of natural disturbances using field and satellite observations.

I confirm that I have read this submission and believe that I have an appropriate level of expertise to confirm that it is of an acceptable scientific standard, however I have significant reservations, as outlined above.
